# Right pulmonary artery agenesis presenting with uncontrolled asthma in an adult: a case report

**DOI:** 10.1186/1752-1947-5-353

**Published:** 2011-08-05

**Authors:** Hafez Hayek, Jaime Palomino, Supat Thammasitboon

**Affiliations:** 1 Section of Pulmonary Diseases, Critical Care and Environmental Medicine. Tulane University Health Sciences Center, 1430 Tulane Ave, SL-9, New Orleans, LA 70123, USA

## Abstract

**Introduction:**

Unilateral absence of the pulmonary artery (UAPA) or pulmonary artery agenesis is a rare congenital disorder presenting with a wide spectrum of symptoms. The clinical presentation is variable and many patients can be asymptomatic for many years and even throughout their lives.

**Case presentation:**

We report the case of a 53-year-old African-American woman who was diagnosed with right pulmonary artery agenesis after presenting with uncontrolled asthma and recurrent bronchopulmonary infections.

**Conclusion:**

In an unexplained case of recurrent respiratory infections and shortness of breath, the possibility of a rare congenital anomaly like UAPA should be considered and an appropriate evaluation should be done.

## Introduction

Unilateral absence of the pulmonary artery (UAPA) or pulmonary artery agenesis is a rare congenital disorder presenting with a wide spectrum of symptoms. The first case was reported in 1868. The prevalence of isolated UAPA is estimated to be around 1 in 200,000 individuals [1]. The clinical presentation is variable and many patients may be asymptomatic for many years and even throughout their lives. Recurrent pulmonary infections, decreased exercise tolerance and shortness of breath on exertion are the most common symptoms. In a literature review by Ten Harkel *et al. *[2], recurrent pulmonary infections were present in 37% of cases, dyspnea or exercise limitations in 40%, hemoptysis in 20%, and high-altitude pulmonary edema in 12%. Pulmonary hypertension was found in 44% of patients which is higher than a previous report of 20-25% [3,4].

## Case presentation

A 53-year-old African-American woman was referred to our pulmonary clinic because of uncontrolled asthma and frequent respiratory infections. Our patient reported frequent asthmatic attacks and symptoms requiring excessive use of rescue inhalers. She also described symptoms consistent with gastroesophageal reflux disorder (GERD) and rhinitis. She had never been a cigarette smoker and her past medical history included hypertension and beta thalassemia. She was also told that she had a congenital vascular abnormality.

Her physical development was normal and there was no family history of congenital cardiovascular disease.

Her asthma treatment regimen included ipratropium bromide and an albuterol inhaler as needed.

At the initial evaluation, our patient was awake and alert, in no acute distress. She was afebrile and her vital signs were stable. Her physical examination revealed decreased breath sounds with mild rhonchi in her right lower lung zone. There were no clinical signs of edema, cyanosis or clubbing of fingers. The rest of the physical examination was unremarkable.

Except for mild anemia, routine hematological and biochemical profiles were within the normal ranges. An allergy skin test was positive for dust mites, cats and multiple grasses. Inhaled corticosteroids and long acting beta-agonists were added as well as proton pump inhibitors, nasal steroids and anti-histamines. Environmental control was also emphasized to our patient.

On subsequent visits, our patient reported improvement in her GERD and rhinitis symptoms but her asthma control was still not optimal.

**Figure 1:**
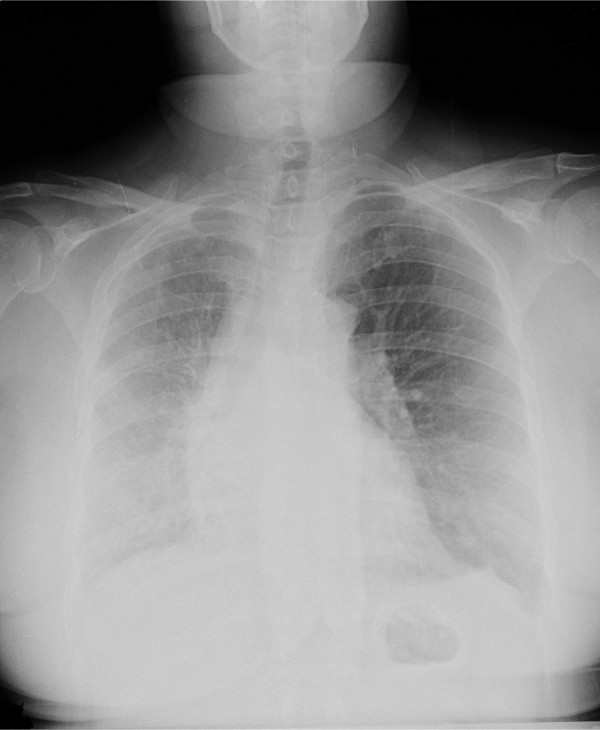
**Chest radiograph showing loss of volume of her right lung with displacement of the mediastinum to the right**.

**Figure 2:**
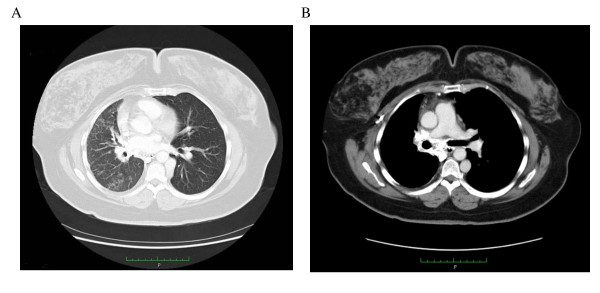
**(A) Chest CT demonstrating volume loss of her right lung associated with increased interstitial markings and displacement of the mediastinum to the right**. **(B) A contrast-enhanced CT scan showed atresia of her right pulmonary artery.**

A plain chest radiograph showed a loss of volume of her right lung and an increased density in her right lower lung zone, with displacement of the mediastinum to the right (Figure 1). A contrast-enhanced computed tomography (CT) of her thorax was performed and revealed absence of the right pulmonary artery with displacement of her heart and mediastinum to the right, and volume loss associated with increased interstitial markings involving her right lung. There was no significant bronchiectasis (Figure 2).

A pulmonary function test showed a forced expiratory volume in one second (FEV1) to forced vital capacity (FVC) ratio of 83%; FEV1 of 1.43 L (65% of predicted), FVC of 1.72 L (64% of predicted), total lung capacity of 67% of predicted and a diffusion capacity for carbon monoxide of 75% of predicted. After a bronchodilator challenge, FVC increased to 2.7 L (102% of predicted) which is consistent with a significant response.

## Discussion

Congenital UAPA is a rare anomaly that may occur in isolation but most frequently is accompanied by cardiovascular malformations such as tetralogy of Fallot, septal defects, patent ductus arteriosus, coarctation of the aorta and transposition of great vessels [5].

The main embryologic defect is an involution of the proximal sixth aortic arch of the affected side, leading to an absence of the proximal pulmonary artery. Intrapulmonary vessels and distal portion of the affected pulmonary artery trunk can develop normally, and blood supply is achieved by systemic collaterals from bronchial, major aortopulmonary collaterals and other systemic arteries [1].

UAPA is twice as common on the right side. However, left-sided agenesis is frequently associated with life-threatening cardiovascular malformations and therefore early diagnosis and surgical repair are required during the first year of life [6,7]. Conversely, patients with isolated right pulmonary artery agenesis survive into adulthood with minimal or no symptoms, making the diagnosis of such cases more challenging. Multiple conditions like Swyer-James-MacLeod's syndrome (SJMS), compensatory emphysema and pulmonary thromboembolic disease can have similar radiographic appearance.

In one report, 30% of patients were asymptomatic [8]. The majority of patients were identified incidentally during routine medical evaluation performed for different reasons [2]. Symptoms can sometimes be unmasked by factors such as pregnancy or high altitude [9]. Due to the nonspecific clinical characteristics of the disease, a delay in diagnosis of 30 years after the onset of symptoms can be observed [2].

Typical chest radiographic findings are ipsilateral cardiac and mediastinal displacement, ipsilateral hemidiaphragm elevation with volume loss of the affected lung, absent hilar shadow and hyperinflation of the controlateral lung [10].

A contrast-enhanced CT of the thorax can confirm the absence of the affected pulmonary artery. High resolution CT scanning can also evaluate the presence of bronchiectasis in cases of recurrent bronchopulmonary infections. Magnetic resonance imaging (MRI) is helpful in the evaluation of congenital cardiovascular defects [11].

Echocardiography is a good tool to establish the diagnosis, to exclude any other cardiac or major vessels abnormalities and to evaluate the presence of associated pulmonary hypertension. Ventilation-perfusion scintigraphy can be useful in the diagnosis and in distinguishing UAPA from SJMS, but this study is limited in its ability to demonstrate pulmonary vascular anatomy and collateral arterial supply [10].

Angiography remains the gold standard for the diagnosis of pulmonary artery agenesis. Currently--with the development of CT, MRI and magnetic resonance angiographic techniques --it is rarely performed unless embolization is indicated for massive hemoptysis [10]. When revascularization is considered, cardiac catheterization should be done with pulmonary venous wedge angiography to visualize hidden pulmonary arteries in the hilum [2].

The treatment of UAPA in adults depends upon the clinical presentation and multiple therapeutic approaches which have been described. In one report [2], 8% of the patients underwent either a pneumonectomy or lobectomy for recurrent hemoptysis or intractable pulmonary infections. When pulmonary hypertension is present, revascularization of the absent artery is recommended and may improve the condition of the patient. If revascularization is not an option or when pulmonary hypertension is not improved, medical treatment described for patients with primary pulmonary hypertension may be helpful.

## Conclusion

Clinicians should be aware of the possibility of undiagnosed cases of UAPA presenting with recurrent respiratory infections. A chest radiograph is usually the initial investigation that suggests the diagnosis. Echocardiography is helpful for the evaluation of possible pulmonary hypertension. Confirmation of the diagnosis and anatomic details can be discerned by CT scanning and MRI. Angiography is reserved for patients requiring embolization or revascularization surgery.

Our patient was diagnosed with UAPA after presenting with recurrent pulmonary infections complicating her asthma course. She refused further invasive workup; she was treated with an oral course of broad-spectrum antibiotics and her symptoms improved after optimizing her asthma therapy.

## Consent

Written informed consent was obtained from the patient for the publication of this case report and accompanying images. A copy of the written consent is available for review by the Editor-in-Chief of this journal.

## Abbreviations

CT: computed tomography; FEV1: forced expiratory volume in one second; GERD: gastroesophageal reflux disorder; FVC: forced vital capacity; MRI: magnetic resonance imaging; SJMS: Swyer-James-MacLeod's Syndrome; UAPA: unilateral absence of the pulmonary artery.

## Competing interests

The authors declare that they have no competing interests.

## Authors' contributions

HH analyzed and interpreted the patient data, searched the relative literature and was a major contributor in writing the manuscript. JP was involved in drafting the manuscript. ST managed the patient and critically revised the manuscript. All authors read and approved the final version of the manuscript.
